# Functional Characterization of miR-216a-5p and miR-125a-5p on Pancreatic Cancer Stem Cells

**DOI:** 10.3390/ijms26072830

**Published:** 2025-03-21

**Authors:** Grazia Fenu, Carmen Griñán-Lisón, Federica Etzi, Aitor González-Titos, Andrea Pisano, Belén Toledo, Cristiano Farace, Angela Sabalic, Esmeralda Carrillo, Juan Antonio Marchal, Roberto Madeddu

**Affiliations:** 1 Department of Biomedical Science, University of Sassari, 07100 Sassari, Italy; grazia.fenu94@gmail.com (G.F.); federicaetzi@gmail.com (F.E.); cristiano.farace@hotmail.it (C.F.); angelasabalic08@gmail.com (A.S.); rmadeddu@uniss.it (R.M.); 2 Department of Biochemistry and Molecular Biology 2, Faculty of Pharmacy, University of Granada, Campus de Cartuja s/n, 18071 Granada, Spain; 3 Centre for Genomics and Oncological Research, GENYO, Pfizer/University of Granada/Andalusian Regional Government, 18016 Granada, Spain; 4 Instituto de Investigación Biosanitaria ibs.GRANADA, University Hospitals of Granada-University of Granada, 18100 Granada, Spain; aitorgt@ugr.es (A.G.-T.); belentoledo@ugr.es (B.T.); esmeral@ugr.es (E.C.); jmarchal@ugr.es (J.A.M.); 5 Excellence Research Unit “Modeling Nature” (MNat), University of Granada, 18100 Granada, Spain; 6 Biopathology and Regenerative Medicine Institute (IBIMER), Centre for Biomedical Research (CIBM), University of Granada, 18100 Granada, Spain; 7 Department of Human Anatomy and Embryology, Faculty of Medicine, University of Granada, 18016 Granada, Spain; 8 National Institute of Biostructures and Biosystems, 00136 Rome, Italy; 9 International Society for Research on Cadmium and Trace Element Toxicity, 07100 Sassari, Italy

**Keywords:** PDAC, CSC, miRNA, functional study, miR-216a-5p, miR-125a-5p, miRNA regulation

## Abstract

Pancreatic ductal adenocarcinoma (PDAC) is the third leading cause of cancer-related death. Its poor prognosis is closely related to late-stage diagnosis, which results from both nonspecific symptoms and the absence of biomarkers for early diagnosis. MicroRNAs (miRNAs) exert a regulatory role in numerous biological processes and their aberrant expression has been found in a broad spectrum of diseases, including cancer. Cancer stem cells (CSCs) represent a driving force for PDAC initiation, progression, and metastatic spread. Our previous research highlighted the interesting behavior of miR-216a-5p and miR-125a-5p related to PDAC progression and the CSC phenotype. The present study aimed to evaluate the effect of miR-216a-5p and miR-125a-5p on the acquisition or suppression of pancreatic CSC traits. BxPC-3, AsPC-1 cell lines, and their CSC-like models were transfected with miR-216a-5p and miR-125a-5p mimics and inhibitors. Following transfection, we evaluated their impact on the expression of CSC surface markers (CD44/CD24/CxCR4), ALDH1 activity, pluripotency- and EMT-related gene expression, and clonogenic potential. Our results show that miR-216a-5p enhances the expression of CD44/CD24/CxCR4 while negatively affecting the activity of ALDH1 and the expression of EMT genes. MiR-216a-5p positively influenced the clonogenic property. MiR-125a-5p promoted the expression of CD44/CD24/CxCR4 while inhibiting ALDH1 activity. It enhanced the expression of *Snail*, *Oct-4*, and *Sox-2*, while the clonogenic potential appeared to be affected. Comprehensively, our results provide further knowledge on the role of miRNAs in pancreatic CSCs. Moreover, they corroborate our previous findings about miR-216a-5p’s potential dual role and miR-125a-5p’s promotive function in PDAC.

## 1. Introduction

Pancreatic ductal adenocarcinoma is one of the most lethal human cancers. The incidence and mortality rates for PDAC are remarkably similar, which underscores its severely malignant nature. Projections for 2024 indicated that there would be approximately 66,440 new diagnoses and 51,750 deaths in the United States directly attributable to PDAC [1]. Although a variety of therapeutic regimens have been implemented in the adjuvant or neoadjuvant setting of PDAC, including single-agent therapies and the combination of cytotoxic compounds such as FOLFIRINOX or nab-paclitaxel, surgical resection remains the only effective therapeutic approach [2,3,4]. PDAC is the third leading cause of cancer-related death, and its dismal prognosis is closely associated with late diagnosis, commonly achieved in the advanced stages of the disease, when patients are no longer eligible for surgical resection [5,6,7].

MicroRNAs are non-coding RNAs with a length of 19–24 nucleotides that act as negative regulators of gene expression. They play a crucial role in various biological processes, and their dysregulation has been associated with numerous malignancies, including PDAC [8,9,10,11]. In cancer, the effect of miRNAs depends on their specific targets, and they can therefore have tumor-promoting (oncomiRs) or tumor-suppressing functions. In the context of PDAC, early diagnosis is crucial to improving therapeutic outcomes and patient survival. In this regard, miRNAs could serve as excellent diagnostic candidates due to their remarkable stability in body fluids and their significant biological roles [9,10,11]. Their presence in circulation, shielded from endogenous RNase activity, makes them particularly suitable for use as biomarkers in non-invasive diagnostic applications [8].

Cancer stem cells share self-renewal and differentiation capabilities with normal stem cells and reside within specialized tumor niches. According to the hierarchical model of carcinogenesis, CSCs are key players in tumor initiation, progression, metastasis, recurrence, and therapy resistance [3,12,13,14]. Pancreatic cancer stem cells (PaCSCs), which constitute less than 1% of all pancreatic cancerous cells, are distinguished by their high expression levels of CD44, EpCAM, CD133, CxCR4, CD24, ALDH1, c-MET, DclK1, and Lgr5. Furthermore, the expression of PaCSC markers varies between high-grade PanIN and advanced PDAC [15,16,17,18,19].

Numerous studies have investigated the role of miRNAs in PaCSCs, revealing their ability to regulate various pathways that affect the CSC phenotype by targeting specific molecules [17,20,21]. We previously enlightened the dramatical dysregulation of miR-216a-5p and miR-125a-5p in CSC-like models [22]. In our former research on ex vivo and in vitro models of PDAC, we found that miR-216a-5p was overexpressed during the early PDAC stages, while its expression decreased in advanced PDAC, and this behavior suggested a potential dual role of the miRNA. In our study, miR-125a-5p performed as an oncomiR, a finding that aligns with previous literature search results. Interestingly, in CSC-like models, the behavior of the two miRNAs varied considerably from what we observed in adherent cell lines.

The intricate network of miRNA-mediated regulation in CSC pathways remains poorly understood, and continued investigation is essential to advance the field of translational medicine for both diagnostic and therapeutic applications. Therefore, this study aimed to investigate the influence of miR-216a-5p and miR-125a-5p on the CSC phenotype by evaluating whether these molecules trigger the acquisition or regression of CSC traits. To this end, we evaluated the effect of miRNA mimic and inhibition on pluripotency and epithelial-to-mesenchymal transition (EMT) gene expression, the levels of PaCSC markers, and the anchorage-independent growth of CSC-like models. Our experimental design involves adherent PDAC cell lines and their CSC-enriched counterparts to provide a comprehensive functional characterization of these miRNAs in the context of pancreatic CSCs.

## 2. Results

### 2.1. miR-216a-5p and miR-125a-5p Effect on CD44/CD24/CxCR4 Expression

**Figure 1:**
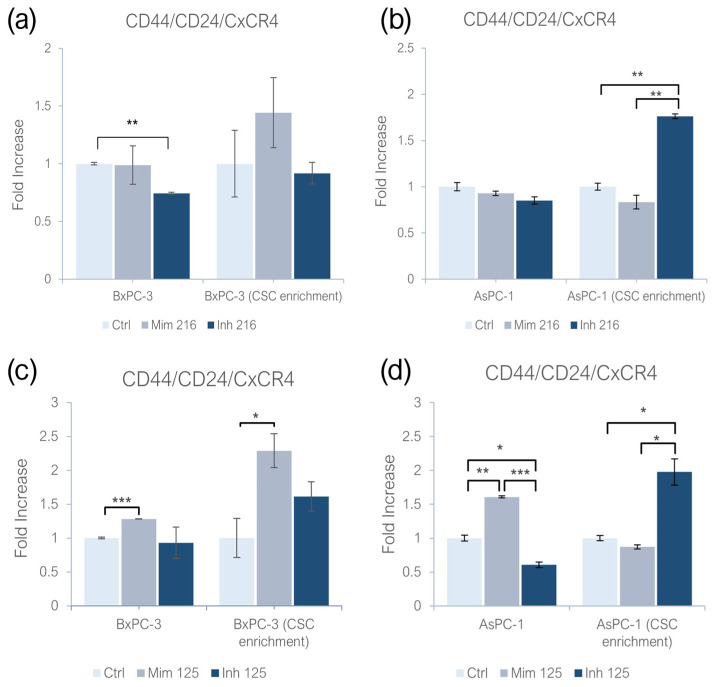
Effect of miR-216a-5p on CD44/CD24/CxCR4 expression in (**a**) adherent BxPC-3 and their CSC-like models; and (**b**) adherent AsPC-1 and their CSC-like models. Effect of miR-125a-5p on CD44/CD24/CxCR4 expression in (**c**) adherent BxPC-3 and their CSC-like models; and (**d**) adherent AsPC-1 and their CSC-like models. Values were normalized with the control cells in monolayer and in CSC enrichment. We attributed different *p*-values to the asterisks: * *p* < 0.05; ** *p* < 0.01; *** *p* < 0.001.

Mimicking the miR-216a-5p effect, both adherent BxPC-3 and AsPC-1 showed no significant changes in CD44/CD24/CxCR4 expression; while inhibiting it, the expression of CSC markers in BxPC-3 was significantly reduced in comparison with the controls (*p* < 0.01). No statistical significant changes were detected on adherent AsPC-1. Investigating the role of this molecule on CSC-like models of BxPC-3, no significant changes were found. In AsPC-1 CSC-like models treated with miR-216a-5p inhibitor, the expression of PaCSC markers increased (*p* < 0.01), while no statistically significant changes were detected following the treatment with the miR-216a-5p mimic (Figure 1a,b). When treating monolayer-grown cell lines with the miR-125a-5p mimic, CD44/CD24/CxCR4 expression increased in both BxPC-3 (*p* < 0.001) and AsPC-1 (*p* < 0.01). Inhibiting miR-125a-5p, the expression of CD44/CD24/CxCR4 decreased in AsPC-1 (*p* < 0.05), while non-statistically significant differences were found on BxPC-3. In BxPC-3 CSC-like models, the effect of miR-125a-5p resulted in an increased expression of PaCSC markers (*p* < 0.05). When treating the same models with the miR-125a-5p inhibitor, no statistically significant changes were detected. In AsPC-1 CSC-like models, the treatment with the miR-125a-5p mimic did not induce statistically significant changes, while the inhibition of the molecule produced an increased expression of CD44/CD24/CxCR4 (*p* < 0.05) (Figure 1c,d).

### 2.2. miR-216a-5p and miR-125a-5p Effect on ALDH1 Activity

**Figure 2:**
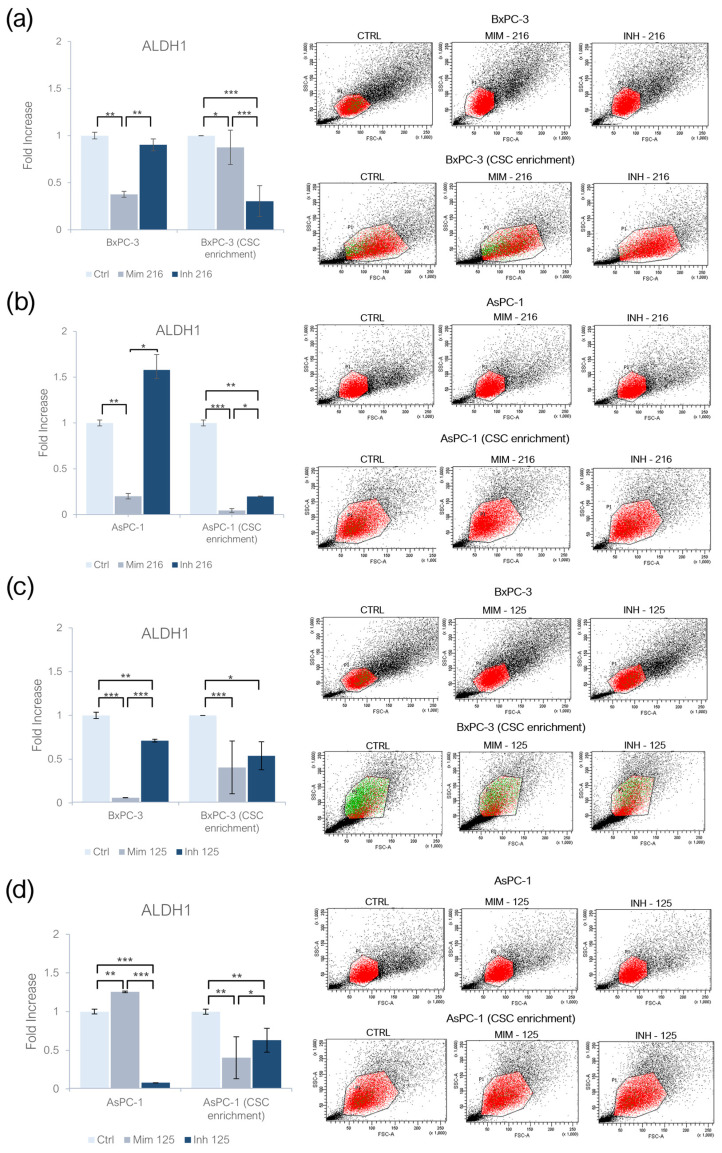
Effect of miR-216a-5p on ALDH1 activity and representative flow cytometry cytogram plot of (**a**) adherent BxPC-3 and their CSC-like models; and (**b**) adherent AsPC-1 and their CSC-like models. Effect of miR-125a-5p on ALDH1 activity and representative flow cytometry cytogram plot in (**c**) adherent BxPC-3 and their CSC-like models; and (**d**) adherent AsPC-1 and their CSC-like models. *x*-axis: FSC-A/ALDH1, *y*-axis: SSC-A/side scatter. Red dots refer to the entire population while green dots are representatives of ALDH1+ population. Values were normalized with the control cells in monolayer and in CSC enrichment. We attributed different *p*-values to the asterisks: * *p* < 0.05; ** *p* < 0.01; *** *p* < 0.001.

The treatment of BxPC-3 with the miR-216a-5p mimic induced a decrease in ALDH1 activity (*p* < 0.01); following the treatment with miR-216a-5p inhibitor, no statistically significant changes were found. In AsPC-1 treated with the miR-216a-5p mimic, ALDH1 activity significantly decreased (*p* < 0.01), while following its inhibition, no significant changes were detected. In CSC-like models built on BxPC-3 cell lines, the treatment with the miR-216a-5p mimic resulted in a decreased activity of ALDH1 (*p* < 0.05), but this behavior was detected also following the inhibition of the molecule: inhibiting miR-216a-5p ALDH1 activity was lower than following the treatment with the mimic (*p* < 0.001). In AsPC-1 CSC-like models, ALDH1 activity decreased after the treatment with the miR-216a-5p mimic (*p* < 0.001) and inhibitor (*p* < 0.01), but, in the latter, the activity was higher when compared to ALDH1 activity in models treated with the mimic (*p* < 0.05) (Figure 2a,b). Following the treatment with the miR-125a-5p mimic, the activity of ALDH1 was decreased in adherent BxPC-3 (*p* < 0.001) and increased in monolayer-grown AsPC-1 (*p* < 0.01), as compared to the controls (Figure 2c,d). In both inhibitor-treated cell lines, ALDH1 activity was lower than in untreated cells, with statistical significance. As compared to the mimic condition, the activity of ALDH1 following the inhibition of miR-125a-5p was higher in BxPC-3 (*p* < 0.001) and lower in AsPC-1 (*p* < 0.001). In CSC-like models derived from BxPC-3 (*p* < 0.001) and AsPC-1 (*p* < 0.01), a decrease in ALDH1 activity was observed as a result of both mimic and inhibitor treatments. In the inhibitor-treated CSC-like models, ALDH1 activity was higher than the one in the mimic-treated models (*p* < 0.05) (Figure 2c,d).

### 2.3. miR-216a-5p Influence on Pluripotency-Related Genes

**Figure 3:**
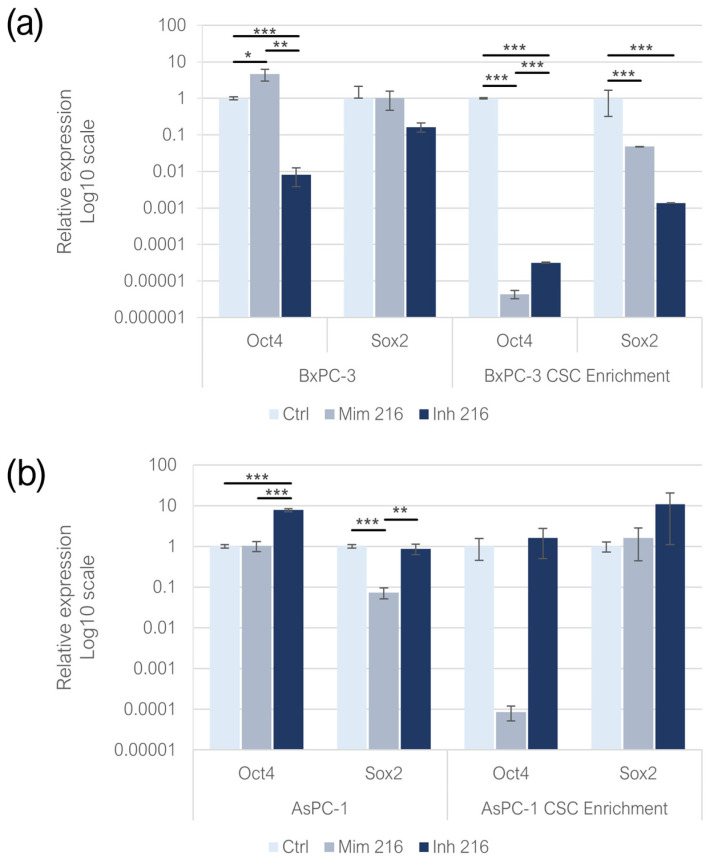
Pluripotency-related gene relative expression following miR-216a-5p mimic and inhibitor treatments on monolayer-grown BxPC-3 and their CSC-like models (**a**) and on monolayer-grown AsPC-1 and their CSC-like models (**b**). We attributed different *p*-values to the asterisks: * *p* < 0.05; ** *p* < 0.01; *** *p* < 0.001.

Enhancing miR-216a-5p in adherent BxPC-3 resulted in an increased expression of *Oct-4* compared to the controls (*p* < 0.05). After the inhibition of miR-216a-5p, the expression of *Oct-4* strongly decreased (*p* < 0.001). The same model treated with the miR-216a-5p mimic and inhibitor exhibited no significant changes in the expression of *Sox-2*. In adherent AsPC-1, the treatment with miR-216a-5p mimic did not induce significant changes in *Oct-4* expression, while, following the inhibition of miR-216a-5p, *Oct-4* expression significantly decreased (*p* < 0.001). In adherent AsPC-1, *Sox-2* expression was decreased compared to the controls following the treatment with the mimic (*p* < 0.001), with non-statistically significant changes in the inhibitor condition. When comparing the expression levels between the mimic and inhibitor conditions, it was found to be overexpressed in the inhibitor condition as compared to the mimic (*p* < 0.01). The expression of *Oct-4* in CSCs derived from the BxPC-3 cell line exhibited a strong reduction in both mimic and inhibitor conditions, compared to the control group (*p* < 0.001): in the inhibitor condition, *Oct-4* was overexpressed compared to cells transfected with the mimic (*p* < 0.001). Regarding the expression of *Sox-2* in the same model, both the treatments induced a downregulation of *Sox-2* compared to the controls (*p* < 0.001): when comparing the mimic and inhibitor conditions, *Sox-2* expression was higher in the mimic compared to the inhibitor condition (*p* < 0.001). In AsPC-1 CSC-like models, the treatment with miR-216a-5p mimic and inhibitor did not induce statistically significant changes in the expression of *Oct-4* and *Sox-2* (Figure 3a,b).

### 2.4. miR-125a-5p Influence on Pluripotency-Related Genes

**Figure 4:**
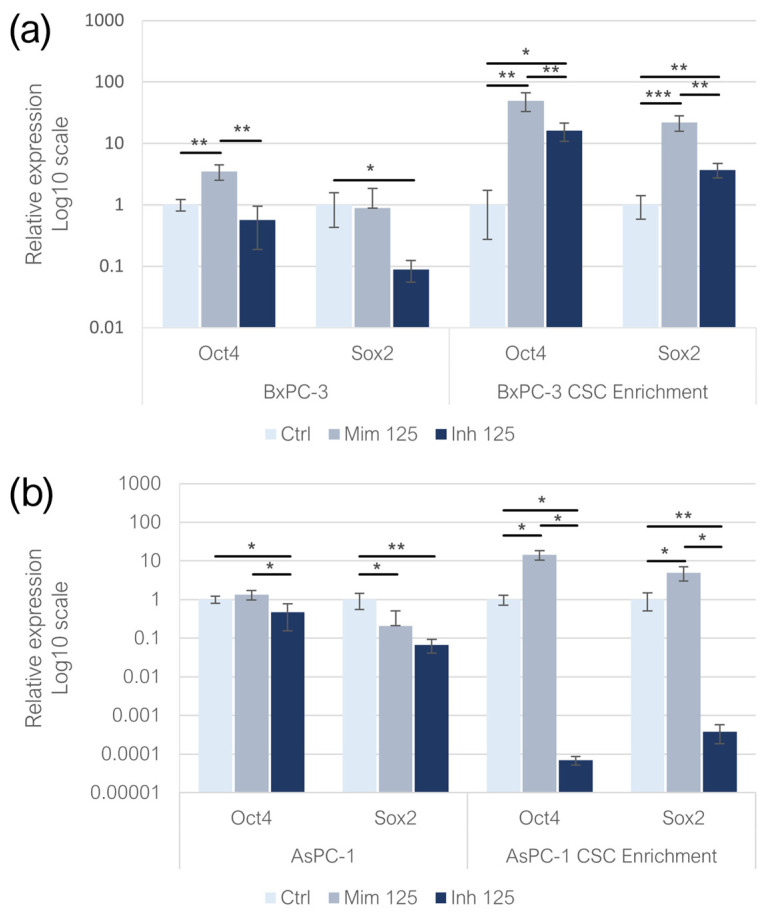
Pluripotency-related gene relative expression following miR-125a-5p mimic and inhibitor treatments on monolayer-grown BxPC-3 and their CSC-like models (**a**) and on monolayer-grown AsPC-1 and their CSC-like models (**b**). We attributed different *p*-values to the asterisks: * *p* < 0.05; ** *p* < 0.01; *** *p* < 0.001.

In adherent BxPC-3 transfected with the miR-125a-5p mimic, the expression of *Oct-4* increased compared to the control group (*p* < 0.01), while, in the treatment with the inhibitor, non-statistical significant changes were detected. In the inhibitor-treated adherent AsPC-1, *Oct-4* expression was reduced (*p* < 0.05). No statistically significant change was found in AspC-1 treated with the miR-125a-5p mimic (Figure 4a,b). In adherent AsPC-1, the treatment with the miR-125a-5p mimic induced a decrease in *Sox-2* expression (*p* < 0.05). The same effect was detected following the inhibitor treatments in monolayer-grown BxPC-3 (*p* < 0.05) and AsPC-1 cell lines (*p* < 0.01). In CSC-like models built on BxPC-3, both treatments resulted in the overexpression of *Oct-4* (*p* < 0.01): the overexpression was higher following the treatment with the miR-125a-5p mimic (*p* < 0.05). Enhancing the miR-125a-5p effect, CSC-derived from AsPC-1 exhibited an increase in *Oct-4* expression (*p* < 0.05), while the inhibition of the miRNA induced a decrease in *Oct-4* levels (*p* < 0.05). In both BxPC-3- (*p* < 0.001) and AsPC-1 (*p* < 0.05)-derived CSC-like models, *Sox-2* was overexpressed in the mimic condition. MiR-125a-5p inhibition resulted in the overexpression of *Sox-2* in BxPC-3 CSC-like models (*p* < 0.01) and in the downregulation of the gene in CSC-enriched AsPC-1 (*p* < 0.01) (Figure 4a,b).

### 2.5. Effect of miR-216a-5p on EMT-Related Genes

**Figure 5:**
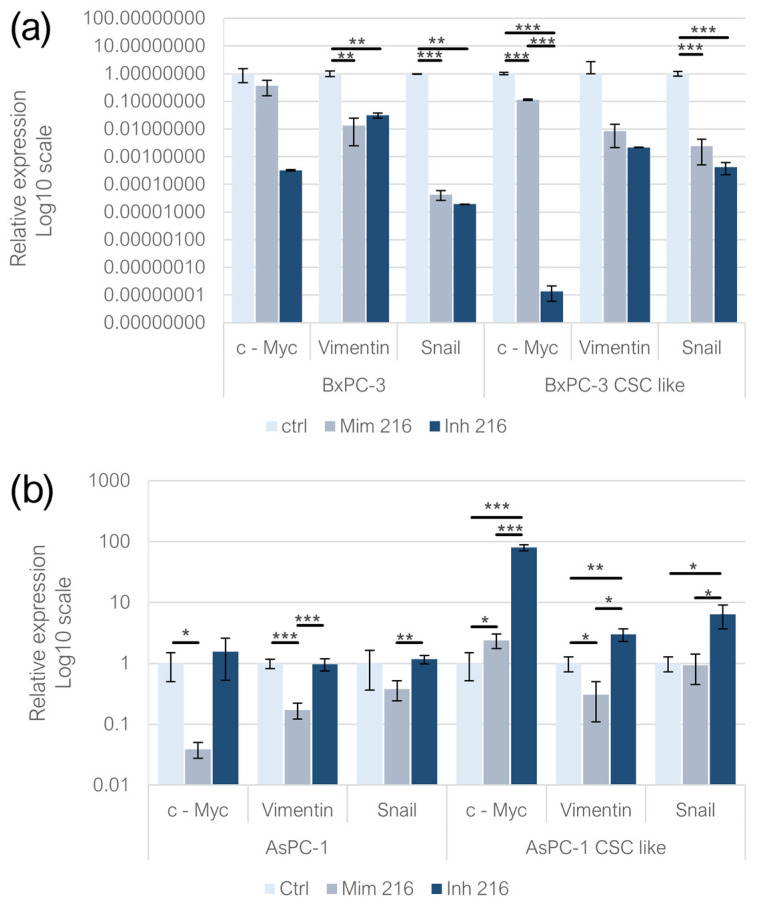
EMT-related gene expression following miR-216a-5p mimic and inhibitor transfection in (**a**) adherent BxPC-3 and CSC-like models and (**b**) in adherent AsPC-1 and their CSC-like models. We attributed different *p*-values to the asterisks: * *p* < 0.05; ** *p* < 0.01; *** *p* < 0.001.

In adherent BxPC-3, the expression of *c-Myc* was not statistically significantly affected by either the mimic and inhibitor treatments. In the BxPC-3-derived CSC-like model, both the mimic and inhibitor treatments resulted in a decreased *c-Myc* expression (*p* < 0.001) compared to the controls; the miR-216a-5p mimic treatment led to higher levels of the gene compared to the inhibitor condition (*p* < 0.001). In adherent AsPC-1 treated with the mimic, the expression of *c-Myc* decreased significantly (*p* < 0.05), while no significant changes were detected following miR-216a-5p inhibition. In CSC-like models built on AsPC-1, both the mimic (*p* < 0.05) and inhibitor (*p* < 0.001) treatments induced an overexpression of *c-Myc*, with a more pronounced trend following miRNA inhibition (*p* < 0.001). The expression of *Vimentin* was affected by the treatment with the miR-216a-5p mimic and inhibitor in adherent BxPC-3 (*p* < 0.01) and following the mimic treatment in monolayer-grown AsPC-1 (*p* < 0.001). In adherent AsPC-1, when comparing the mimic and inhibitor conditions, *Vimentin* levels were higher following the inhibition of miR-216a-5p (*p* < 0.001). No statistically significant changes were detected in BxPC-3-derived CSC-like models, while, in AsPC-1 CSC-like models, the mimic of miR-216a-5p resulted in a decrease in *Vimentin* expression (*p* < 0.05), whereas the inhibition of the miRNA induced the overexpression of the EMT gene (*p* < 0.01). Both the miR-216a-5p mimic (*p* < 0.001) and inhibitor (*p* < 0.01) resulted in *Snail* downregulation in adherent BxPC-3 cells and their CSC-like model (*p* < 0.001). In adherent AsPC-1 cells, neither the miR-216a-5p mimic nor inhibitor treatments resulted in statistically significant changes in *Snail* expression. Nonetheless, a downregulation of *Snail* was observed in the mimic-treated condition compared to the inhibitor-treated condition (*p* < 0.01). In CSC-like models derived from AsPC-1 cells and treated with the miR-216a-5p inhibitor, *Snail* expression was upregulated compared to the controls (*p* < 0.05). Regarding the comparison between the mimic and inhibitor conditions, the observed trend was consistent with that of monolayer-grown AsPC-1 cells (*p* < 0.05) (Figure 5).

### 2.6. Effect of miR-125a-5p on EMT-Related Genes

**Figure 6:**
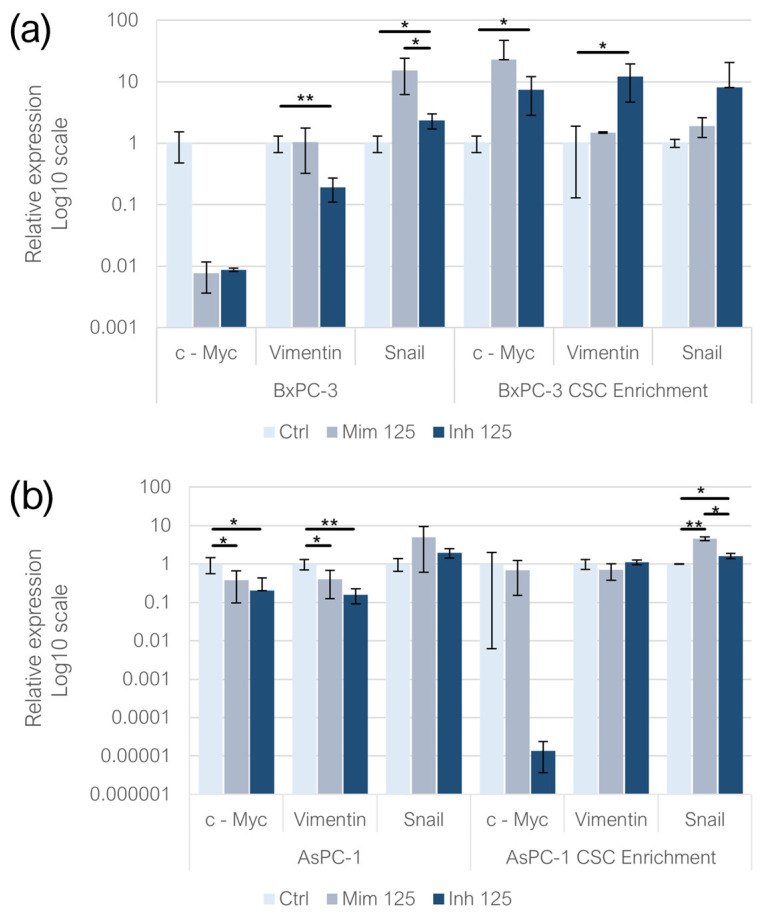
EMT-related gene expression following miR-125a-5p mimic and inhibitor transfection in (**a**) adherent BxPC-3 and CSC-like models and (**b**) AsPC-1 and CSC-like models. We attributed different *p*-values to the asterisks: * *p* < 0.05; ** *p* < 0.01.

In adherent BxPC-3, no statistically significant changes were detected following both the miR-125a-5p mimic and inhibitor treatments. In BxPC-3-derived CSC-like models, miR-125a-5p inhibition induced the overexpression of *c-Myc* (*p* < 0.05). In adherent AsPC-1, the two treatments induced the downregulation of *c-Myc* (*p* < 0.05). No statistically significant changes were detected in CSC-like models derived from AsPC-1. In adherent BxPC-3, *Vimentin* expression was significantly reduced following miR-125a-5p inhibition (*p* < 0.01), whereas no statistically significant changes were observed in BxPC-3-derived CSC-like models. In the AsPC-1 cell line, both the mimic (*p* < 0.05) and inhibitor (*p* < 0.01) treatments led to a reduction in *Vimentin* expression compared to the controls. However, in the CSC-like models derived from AsPC-1, variations in *Vimentin* expression levels were not statistically significant. Regarding *Snail* expression, its levels increased in BxPC-3 cells treated with the miR-125a-5p inhibitor (*p* < 0.05) compared to the controls, while no statistically significant changes were observed following the miR-125a-5p mimic treatment. Similarly, no statistically significant variations were detected in CSC-like models derived from BxPC-3. In monolayer-grown AsPC-1 cells, *Snail* expression variations were not statistically significant. Conversely, in CSC-like models built on AsPC-1, the treatment with both the miR-125a-5p mimic (*p* < 0.01) and inhibitor (*p* < 0.05) increased *Snail* expression levels compared to the controls and, comparing the two conditions, it was higher in the mimic one (*p* < 0.05) (Figure 6).

### 2.7. The Role of miR-216a-5p in Clonogenic Activity

**Figure 7:**
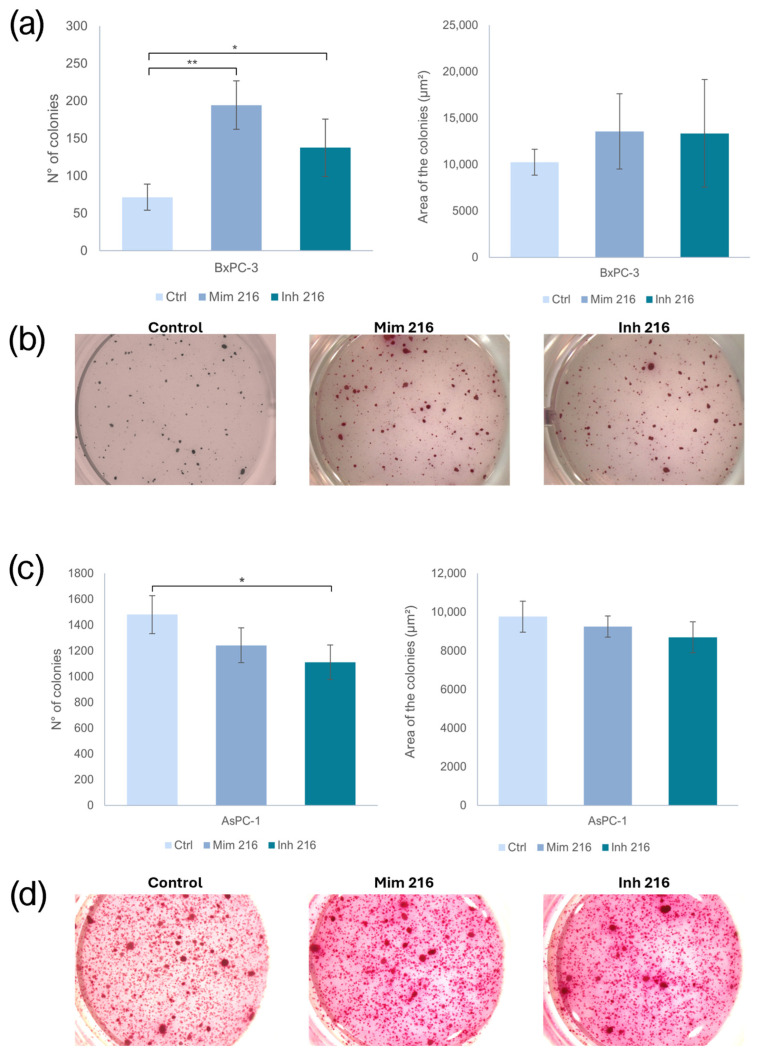
(**a**) Effect of miR-216a-5p on the number and size of BxPC-3 colonies; (**b**) representative images of untreated BxPC-3 and miR-216a-5p mimic-treated and miR-216a-5p inhibitor-treated BxPC-3 in the soft-agar colony formation assay; (**c**) effect of miR-216a-5p on the number and size of AsPC-1 colonies; (**d**) representative imagines of untreated AsPC-1 and miR-216a-5p mimic-treated and miR-216a-5p inhibitor-treated AsPC-1 in the soft-agar colony formation assay. We attributed different *p*-values to the asterisks: * *p* < 0.05; ** *p* < 0.01.

The treatment of BxPC-3 CSC-like PaCSCs with the miR-216a-5p mimic induced an increased number of colonies when compared to the control (*p* < 0.01). The PaCSCs treated with the inhibitor of miR-216a-5p showed a higher number of colonies than the one in the controls (*p* < 0.05). However, the area of the colonies did not change in a statistically significant manner. Following miR-216a-5p inhibition, the AsPC-1 CSC-like models showed fewer colonies than the controls (*p* < 0.05). Concerning the area of the colonies, no statistically significant change was found (Figure 7).

### 2.8. The Role of miR-125a-5p in Clonogenic Activity

**Figure 8:**
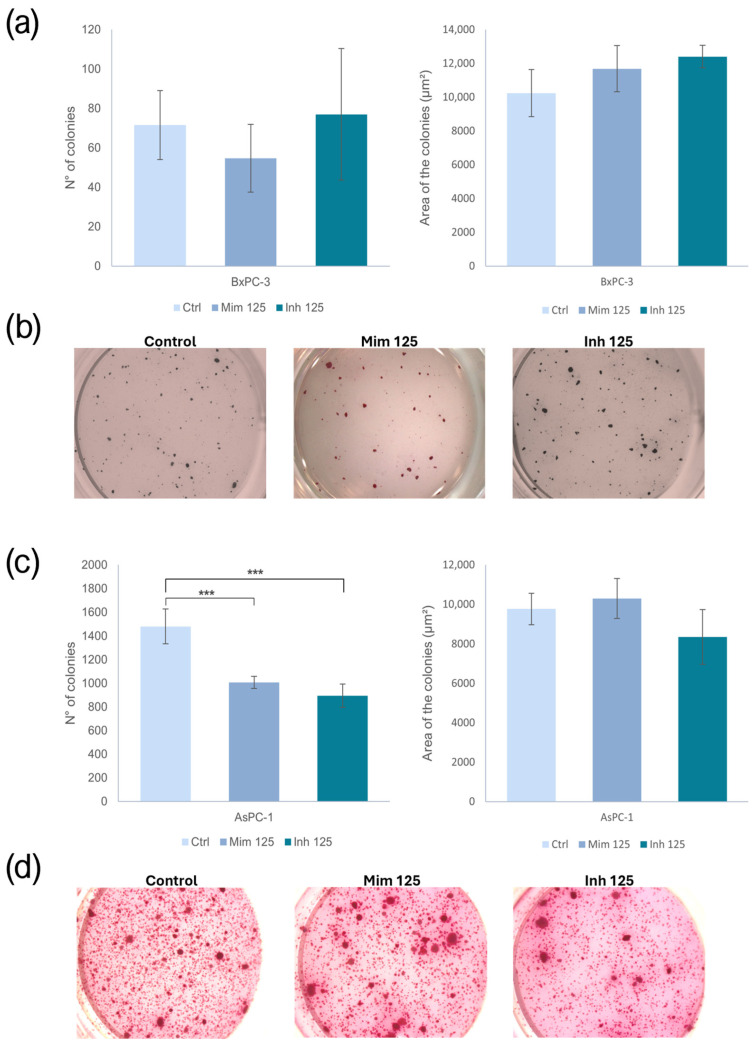
(**a**) Effect of miR-125a-5p on the number and size of BxPC-3 colonies; (**b**) representative images of untreated BxPC-3 and miR-125a-5p mimic-treated and miR-125a-5p inhibitor-treated BxPC-3 in the soft-agar colony formation assay; (**c**) effect of miR-125a-5p on the number and size of AsPC-1 colonies; (**d**) representative images of untreated AsPC-1 and miR-125a-5p mimic-treated and miR-125a-5p inhibitor-treated AsPC-1 in the soft-agar colony formation assay. We attributed different *p*-values to the asterisks: *** *p* < 0.001.

Enhancing the miR-125a-5p effect on BxPC-3 CSC-like models, the number and the area of the colonies did not exhibit statistically significant changes. In AsPC-1 CSC-like models, the treatment with the miR-125a-5p mimic resulted in fewer colonies compared to the controls (*p* < 0.001). The same behavior was observed following the treatment with the miR-125a-5p inhibitor (*p* < 0.001). No statistically significant variations were reported in the area of the colonies, following both the mimic and inhibitor treatments (Figure 8).

## 3. Discussion

The current study builds upon our previous research regarding the involvement of miRNAs in PDAC [22]. Our prior findings enabled us to focus on miR-216a-5p and miR-125a-5p for functional investigation. Multiple research efforts have documented the downregulation of miR-216a-5p in PDAC cases, and various intricate regulatory mechanisms have been proposed to elucidate its potential tumor-suppressive role [23,24,25,26]. The involvement of miR-125a-5p in cancer has been extensively investigated, with often contradictory findings. Despite this, its role in PDAC remains poorly explored [27]. Given our previous results reporting a significant deregulation of these miRNAs in PaCSC models and considering the critical role of CSCs in tumorigenesis, progression, and metastasis, we deemed it necessary to evaluate the effects of miR-216a-5p and miR-125a-5p on CSC trait acquisition or regression [22]. To this end, we analyzed their impact on the expression of stemness markers, pluripotency, and EMT genes in adherent BxPC-3 and AsPC-1 and their CSC-enriched spheres, as well as in the anchorage-independent growth of PaCSC models.

It has been reported that, in physiological conditions, miR-216a-5p is abundant in pancreatic acinar cells and that its levels could vary following cellular degeneration and necrosis [28,29]. In PDAC, miR-216a-5p levels have been frequently found to be downregulated and it has been described as a tumor-suppressive miRNA. Furthermore, Felix et al. reported that the molecule targets *IL-6*, a cytokine whose levels are frequently increased in PDAC and associated with poor prognosis. Despite extensive research on the role of miR-216a-5p in PDAC and other types of cancer, its potential connection with PaCSCs and their associated markers CD44/CD24/CxCR4 and ALDH1 remains unexplored. In breast cancer, miR-216a-5p has been reported as a negative regulator of EMT, ALDH expression, and stemness genes and has been demonstrated that it exerts its role by educating the tumoral microenvironment through cytokine production [23,24,30,31]. In our study, miR-216a-5p promoted the expression of CD44/CD24/CxCR4 in both adherent BxPC-3 and their CSC-enriched spheres, and a trend of positive influence was also identified in AsPC-1 CSC-like models. Nevertheless, ALDH1 expression was affected by miR-216a-5p. The effects of miR-216a-5p and miR-125a-5p on CSC markers, ALDH1 activity, and EMT-related genes may be mediated through key signaling pathways. miR-216a-5p has been reported to influence the *JAK2/STAT3* pathway, which plays a crucial role in CSC regulation and EMT progression [32]. Additionally, miR-125a-5p may modulate *STAT3* activity, potentially affecting pluripotency and ALDH1 expression [33]. These findings suggest that both miRNAs contribute to the modulation of CSC properties through their interaction with fundamental oncogenic pathways.

These regulatory mechanisms align with the broader pathways that govern CSC traits and EMT progression. EMT enhances CSC traits, increasing invasiveness and resistance to apoptosis [34,35]. This process is regulated by key pathways such as *TGF-β* and *Wnt/β-catenin*, which modulate transcription factors like Snail and SOX2, promoting tumor aggressiveness [34,36]. Targeting the CSC-EMT axis may help reduce metastasis and therapeutic resistance [34,36]. Our results on the AsPC-1 cell line show that miR-216a-5p inhibited *c-Myc*, *Snail*, and *Vimentin* expression. In BxPC-3, this behavior was mirrored for *Vimentin*. The negative influence of miR-216a-5p on EMT has already been described in prostate cancer, where the overexpression of the miRNA reduced *N-cadherin*, *Vimentin*, and *Snail* levels [37]. Furthermore, we observed that the effect of miR-216a-5p on pluripotency genes changed depending on the cell lines and the phenotypes considered: only in adherent BxPC-3 miR-216a-5p seemed to exert a promotive influence, while in BxPC-3 enriched in CSCs and in adherent and CSC-like AsPC-1, the miRNA had an inhibitory effect. The differences in the role of miRNA between the two cell lines and their phenotypes may also originate in their different ability to metastasize [38]. Indeed, whereas the BxPC-3 cell line is representative of the primary tumor and has low metastatic activity, the AsPC-1 cell line is representative of metastatic PDAC and is characterized by high metastatic activity. Although further studies are needed, these findings lead us to think that miR-216a-5p may exert an inhibitory role in cells with a greater malignancy. Concerning the anchorage-independent growth, miR-216a-5p exhibited a promoting influence on the number and size of the colonies of both cell lines. Our previous findings showed that the expression of miR-216a-5p in PDAC may change as the disease evolves, probably suggesting a shift from a promoting function in the early stages to a suppressive role during advanced disease [22]. Our observation is coherent with those of Petrovic et al., who in their study showed how the expression levels of a miRNA can vary during tumor progression, following a time profile [39]. Even if not with miR-216a-5p, the same phenomenon has also been described by Xiang et al. and Ota et al., who enlightened how members of the miRcluster miR-17/92 can simultaneously act as oncogenes and as tumor suppressors: it has been proposed that these molecules try to maintain equilibrium by inhibiting the translation of pro-proliferative and anti-proliferative genes; consequently, an imbalance in their expression levels, in both directions, may induce tumorigenesis [39,40,41]. The results of this functional study agree with our previous hypothesis concerning a potential dual behavior of miR-216a-5p in PDAC. Indeed, we have observed that its effect may vary depending on the models and phenotypes considered. In our opinion, this may suggest how the role of miR-216a-5p may vary according to the grade of malignancy of the model considered and thus the progression of the disease. However, further studies are needed to better clarify it.

Previous studies demonstrated that miR-125a-5p levels were upregulated in CD133^+^ cells from patients with hepatocellular carcinoma compared to the control group. Furthermore, it has been demonstrated that this molecule exhibits an antiapoptotic effect on hematopoietic stem cells [42,43]. Our findings suggest that miR-125a-5p may stimulate the expression of CD44/CD24/CxCR4; it happened in all the cellular models analyzed, with the sole exception of AsPC-1-derived CSC-enriched spheres. The miRNA exerted an inhibitory trend on ALDH1 expression.

The existing literature concerning the potential impact of miR-125a-5p on EMT is rather heterogeneous [44,45]. In their research, Chen et al. demonstrated that miR-125a-5p promotes PDAC progression by activating the *ERK/EMT* signaling pathway [46]. In colorectal cancer, Zhu et al. demonstrated that miR-125a-5p enhances the EMT process through the targeting of *DDB2*, an EMT suppressor [47]. Nevertheless, it has been reported that, in esophageal squamous cell carcinoma, miR-125a-5p inhibits the EMT by targeting *Stat3* and it resulted in enhanced cytotoxicity of cisplatin [48]. Our results show that miR-125a-5p exhibits an inhibitory trend on *Vimentin* expression in both adherent and CSC-like models while enhancing *Snail* expression in the same conditions. In agreement with our observation on EMT genes, it has been demonstrated that, in liver cancer, miR-125a-5p overexpression negatively affects *N-cadherin*, *p53*, *Vimentin*, and *VEGF* expression [49]. The differences in the effect that miR-125a-5p exhibits on *Snail* and *Vimentin* may be related to the interaction between the miRNA and different EMT-related pathways. In this regard, the complex regulatory network underlying the EMT involves various elements, including transcription factors, co-transcription factors, microRNAs and epigenetic modifying enzymes. The regulation that these molecules exert on the EMT depends on and varies according to the interaction between the molecules themselves and the context in which they act [50].

Additionally, miR-125a-5p upregulated *Oct-4* and *Sox-2* expression across all cell models. Its effect on clonogenic potential was particularly striking: in BxPC-3 CSC-like models, it decreased the number of colonies but increased their size. A similar trend was observed in AsPC-1 CSC-like models, suggesting that miR-125a-5p may suppress clonogenicity but not colony expansion.

## 4. Materials and Methods

### 4.1. Experimental Design

The in vitro models of PDAC selected for the study comprised the cell lines BxPC-3, which is representative of the primary tumor, and AsPC-1, which serves as a model for metastatic PDAC. Additionally, the BxPC-3 and AsPC-1 cell lines were cultured in a sphere-forming medium to generate the respective CSC-enriched model. To assess the impact of miR-216a-5p and miR-125a-5p on the CSC phenotype, the adherent cell lines and CSC-like models were transfected with either the miRNA mimic or inhibitor, focusing on a single miRNA at a time. Following transfection, the expression of PaCSC markers was assessed in all experimental conditions, along with the expression of pluripotency genes and EMT. In addition, the anchorage-independent growth of CSC-enriched models was evaluated.

### 4.2. Cell Lines

The cell lines BxPC-3 (ATCC CRL-1687) and AsPC-1 (ATCC CRL-1682) were purchased from ATCC and cultured following the manufacturer’s instructions. These in vitro models of PDAC were used to represent the primary tumor (BxPC-3) and metastatic PDAC (AsPC-1). Both cell lines were grown in RPMI 1640 Medium with ATCC modification (Gibco™, Thermo Fisher Scientific, Waltham, MA, USA) supplemented with 10% of FBS. The cells were maintained in the incubator under standard conditions at 37 °C with 5% CO_2_.

### 4.3. CSC Enrichment

To acquire a subpopulation of pancreatic spheres enriched in CSC, the BxPC-3 and AsPC-1 cell lines were cultured following the patented protocol WO2016020572A1 [22,51,52,53,54]. To obtain the spheres, the protocol was carried out using Ultra-Low Attachment Six-Well Plates (Corning, Costar, AZ, USA) with DMEM F/12 without FBS and supplemented with the following components: 1% penicillin/streptomycin (Pen-Strep-0781; Sigma-Aldrich, St. Louis, MO, USA), 4 ng/mL heparin (Sigma-Aldrich, Merck KGaA, Darmstadt, Germany), 1 µg/mL hydrocortisone (Sigma-Aldrich), 10 µg/mL insulin (Insulin–Transferrin–Selenium, Invitrogen, Thermo Fisher Scientific, Waltham, MA, USA), and 1 × B27 (B-27™ Supplement [50×], Vitamin A; Invitrogen, Carlsbad, CA, USA). Before cell seeding, the CSC medium was supplemented with 10 ng/mL of Epidermal Growth Factor (Sigma-Aldrich), 10 ng/mL of Interleukin-6 (Miltenyi, Bergisch Gladbach, Germany), 10 ng/mL Hepatocellular Growth Factor (Miltenyi), and 10 ng/mL of Fibroblast Growth Factor (Sigma-Aldrich). After 72 h, primary spheres were separated using trypsin or a syringe. Following disaggregation, the cells were re-seeded in low-attachment multi-well plates in sphere-conditioned medium for an additional 72 h, before further use.

### 4.4. Transient Transfection with miR-216a-5p and miR-125a-5p

The transfection of adherent BxPC-3 and AsPC-1 cell lines and their respective CSC-like models was carried out using the transfection reagent TransIT-X2 Dynamic Delivery System (Mirus Bio–Madison, WI, USA) in Opti-MEM I Reduced-Serum Medium (Gibco™, New York, NY, USA). To mimic and inhibit the two molecules, miR-216a-5p miRCURY LNA miRNA Mimic, miR-125a-5p miRCURY LNA miRNA Mimic, miR-216a-5p miRCURY LNA miRNA Inhibitor, and miR-125a-5p miRCURY LNA miRNA Inhibitor were purchased from Qiagen (Hilden, Germany) and used according to the manufacturer’s instructions. For every experiment, a mock control was included. The specificity of transfection was evaluated through the use of Negative control A and Negative Control miRCURY LNA miRNA Mimic as the controls. The transfection efficiency was evaluated by means of the appropriate 5’ FAM-labeled negative controls purchased from Qiagen (Hilden, Germany). Adherent BxPC-3 and AsPC-1 were seeded 18–24 h before transfection, while, for CSC-like models, cell seeding was carried out on secondary spheres. MiRNA mimic and inhibitor complexes were added with Opti-MEM at final concentrations of 5 nM and 50 nM per well, respectively, along with 1.5 µL and 1 µL of TransIT-X2. After an incubation period of 15–30 min, TransIT-X2:miRNA complexes were distributed to the plated cells. After 72 h, cells were harvested and used for the analysis. The transfection efficiency was evaluated using the Negative Control miRCURY LNA miRNA Mimic—5’ FAM (Qiagen, Hilden, Germany) for the mimic treatment and the Negative control A—5’ FAM for the inhibitor one. For the transfections on CSC-like models, the same procedure was followed, but the number of cells seeded was 2.5–5.0 × 10^5^ cells in each well and the volume of transfection reagent used was 2 µL for both treatments. To confirm the success of the transfection, we performed a qPCR analysis of miR-216a-5p and miR-125a-5p expression levels in both the BxPC-3 and AsPC-1 cell lines, following the manufacturer’s instructions (Qiagen), with the miRCURY LNA™ SYBR^®^ Green PCR Kit. (Qiagen, Hilden, Germany). The results demonstrate effective transfection, as shown in Supplementary Figure S1.

### 4.5. Cytofluorimetric Assays for PaCSC Markers

The ALDEFLUOR™ kit assay (Stem Cell Technologies, Vancouver, WA, Canada) was used following the manufacturer’s instructions to identify cells that, as CSCs, express high levels of ALDH1 enzyme. The cell-surface levels of CD24, CD44, and CxCR4 were detected using anti-human CD24 antibody conjugated with phycoerythrin (PE), anti-human CD44 antibody conjugated with fluorescein isothiocyanate (FITC), and anti-human CxCR4 (CD184) conjugated with allophycocyanin (APC). Antibodies were purchased from (Miltenyi Biotec, Bergisch Gladbach, Germany) and used following the manufacturers’ instructions. Briefly, cells were washed, and the pellet was resuspended in 98 µL of PBS/BSA/EDTA buffer with the addition of 2 µL of antibody. The PBS/BSA/EDTA buffer was obtained by diluting the MACS BSA Stock Solution (Miltenyi Biotec) 1:20 with autoMACS^®^ Rinsing Solution (Miltenyi Biotec). The recommended antibody dilution is 1:50 for up to 10^6^ cells in a final volume of 100 µL. Then, the suspension was incubated for 10 min at 2–8 °C in the dark. Following the incubation period, the cells were washed and resuspended in the analysis buffer. Data were normalized to their respective control groups, ensuring comparability across experimental conditions.

### 4.6. RNA Extraction and Retrotranscription

Cells were homogenized using 1000 µL of TRIZOL (Sigma-Aldrich) reagent. Following an incubation period of 15 min and the addition of 200 µL of chloroform, the tube was shaken vigorously and placed at room temperature for 10 min. At the end of this period, the suspension was centrifuged at 4 °C for 15 min and then the upper phase was transferred into a new tube. Following the addition of 500 µL of isopropanol, the tube was vortexed and incubated at room temperature for 10 min. It was centrifuged at 12,000× *g* for 10 min, at 4 °C. Then, the supernatant was eliminated, and 1000 µL of ethanol 75% were added. The tube was shaken vigorously with a vortex and centrifuged at 4 °C, 17,000× *g* for 5 min. The supernatant was discarded, and the pellet was dried. Finally, the pellet was resuspended in 20 µL of nuclease-free water. The quantity of RNA extracted was evaluated using the Nanodrop (NanoDrop™ 2000/2000c Spectrophotometers, Thermo Scientific ™). To proceed with reverse transcription, the kit GoScript™ Reverse Transcriptase (Promega, Madison, WI, USA) was used according to the manufacturer’s instructions. First, the extracted RNA was diluted and submitted to a first reverse transcription cycle, at 70 °C for 10 min. Then, to the RNA, 10 µL of the RT mix was added, composed as follows: MgCl_2_, reaction buffer, dNTPs, primers, enzyme (AMV), and ribonuclease inhibitors. Then, the second reverse transcription cycle was carried out for 30 min.

### 4.7. Real-Time PCR

The cDNA was diluted to a final concentration of 5 ng/µL. The influence of miR-216a-5p and miR-125a-5p on stemness gene expression was evaluated with commercial primers for Nanog, Sox2, and OCT-4, while the effect of the molecules on the EMT process was investigated by means of c-Myc, Vimentin, and Snail expression. The reactions were prepared using SYBR Green PCR Master Mix (Promega), and human GAPDH was used as a reference gene. Each reaction was performed in triplicate. The real-time PCR was run with the StepOne Real-Time PCR System (Thermo Fisher, Waltham, MA, USA) with the following cycling conditions: 95 °C for 2 min (PCR initial heat activation), 95 °C for 10 s (denaturation), and 56 °C for 60 s (combined annealing/extension) for 40 cycles. Data were analyzed using the 2^−∆∆Ct^ method and statistically evaluated with a two-sided non-paired student’s *t* test, considering results with a *p*-value < 0.05 as significant.

### 4.8. Soft-Agar Colony Formation Assay

To study the colony formation capacity, we disaggregated and seeded 20,000 secondary CSCs in 0.4% cell agar base layer with DMEM (Sigma-Aldrich, St. Louis, MO, USA) in a 24-well culture plate, which was on top of a 0.8% base agar layer. We refreshed them every 5 days with DMEM (Sigma-Aldrich, St. Louis, MO, USA). Cells were then incubated for a further 21 days at 37 °C and 5% CO_2_. Cell colony formation was then counted under a light microscope after staining with 1 mg/mL iodonitrotetrazolium chloride (Sigma-Aldrich, St. Louis, MO, USA) overnight at 37 °C. Then, the wells were washed with PBS 1×. The count and the analysis of the colonies were carried out using a dissecting microscope and the ImageJ software (version number 1.54d).

### 4.9. Statistical Analysis

The reported results are all inferred from at least three replications and are expressed as mean ± standard deviation. Statistical significance was obtained by applying a two-sided unpaired Student’s test. Results with *p* < 0.05 were deemed significant.

## 5. Conclusions

Our results highlight the impact of miR-216a-5p and miR-125a-5p on adherent pancreatic tumor cell lines, as well as their CSC-enriched models. Compared to our initial hypothesis, the observed results indicate that miR-216a-5p may have a dual behavior during PDAC progression, but further studies are needed to characterize it. Additionally, our evidence suggests that miR-125a-5p fosters disease progression, through the promotion of the CSC phenotype. A noteworthy limitation is that the results obtained for each miRNA do not respond exclusively to the mimic or inhibitor treatments but are also affected by the distinct mutational profiles of the two cell lines. Nevertheless, our findings contribute to clarifying the regulatory role of miRNAs in the complex PDAC scenario, particularly in PaCSCs, closely related to progression and metastasis. We strongly believe that investigating the involvement of miRNAs in the CSC phenotype may pose the basis for the development of targeted therapies against this subset of cancer cells and their related pathways.

## Data Availability

Data are contained within the article and Supplementary Materials.

## Supplementary Materials

The following supporting information can be downloaded at: https://www.mdpi.com/article/10.3390/ijms26072830/s1.

## Abbreviations

The following abbreviations are used in this manuscript:

miRNA	microRNA
CSC	Cancer Stem Cell
PDAC	Pancreatic Ductal Adenocarcinoma
PaCSCs	Pancreatic Cancer Stem Cells
EMT	Epithelial–Mesenchymal Transition
