# Supplementary material for: Functional Characterization of miR-216a-5p and miR-125a-5p on Pancreatic Cancer Stem Cells
Source: Int J Mol Sci. 2025 Mar 21;26(7):2830. doi: 10.3390/ijms26072830 (PMC11988779; doi:10.3390/ijms26072830)
Supplement: Supplementary file 1 [file ijms-26-02830-s001.zip › ijms-3472520-supplementary.pdf]

Supplementary Figure S1

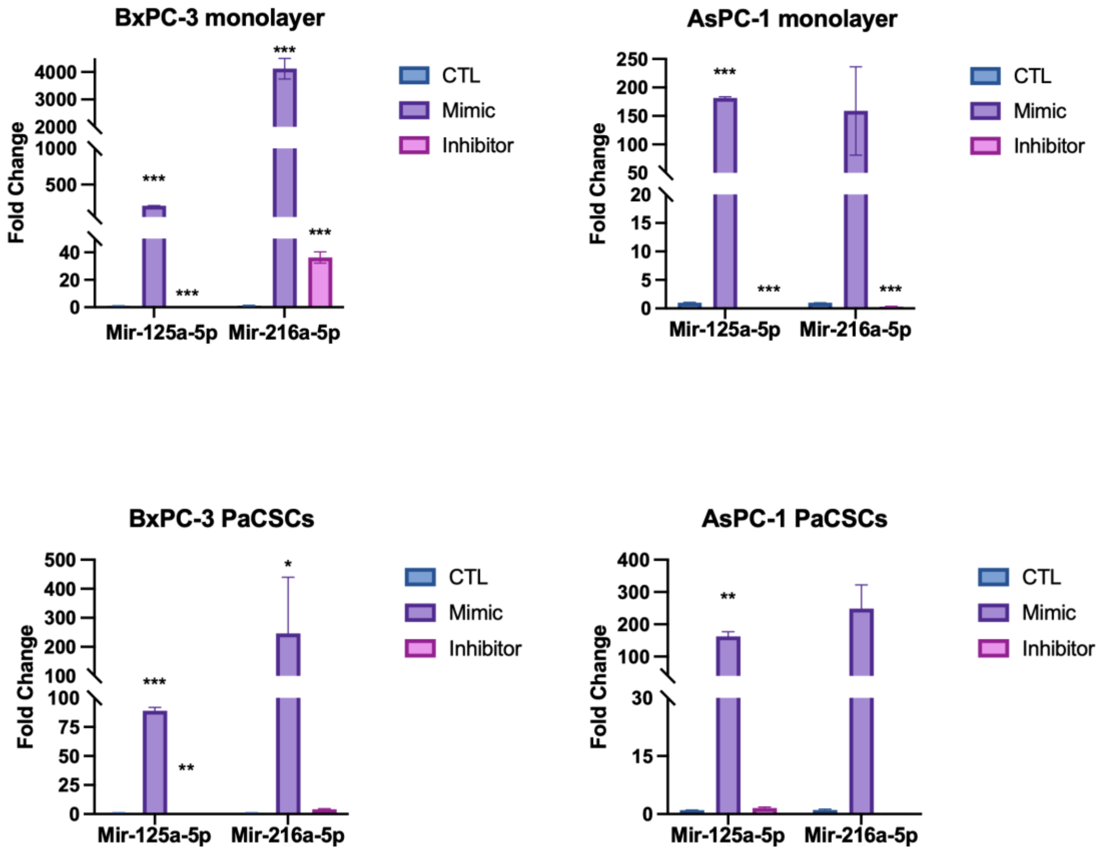

Figure S1. miR-125a-5p and miR-216a-5p relative expression of control, mimic and inhibitor treatment on monolayer and PaCSCs of BxPC-3 and AsPC-1 cell lines. We have attributed different p-values to the asterisks: *$p<0.05$; **$p<0.01$; ***$p<0.001$.
